# Label-free cell phenotypic profiling decodes the composition and signaling of an endogenous ATP-sensitive potassium channel

**DOI:** 10.1038/srep04934

**Published:** 2014-05-12

**Authors:** Haiyan Sun, Ying Wei, Huayun Deng, Qiaojie Xiong, Min Li, Joydeep Lahiri, Ye Fang

**Affiliations:** 1 Biochemical Technologies, Science and Technology Division, Corning Incorporated, Corning, NY 14831, United States of America; 2 The Solomon H. Snyder Department of Neuroscience and High Throughput Biology Center, Johns Hopkins University School of Medicine, Baltimore, Maryland 21205, United States of America; 3 Current address: Biodesign Institute, Arizona State University, AZ 85287, USA; 4 Current address: Cold Spring Harbor Laboratory, Cold Spring Harbor, NY 11724, United States of America

## Abstract

Current technologies for studying ion channels are fundamentally limited because of their inability to functionally link ion channel activity to cellular pathways. Herein, we report the use of label-free cell phenotypic profiling to decode the composition and signaling of an endogenous ATP-sensitive potassium ion channel (K_ATP_) in HepG2C3A, a hepatocellular carcinoma cell line. Label-free cell phenotypic agonist profiling showed that pinacidil triggered characteristically similar dynamic mass redistribution (DMR) signals in A431, A549, HT29 and HepG2C3A, but not in HepG2 cells. Reverse transcriptase PCR, RNAi knockdown, and K_ATP_ blocker profiling showed that the pinacidil DMR is due to the activation of SUR2/Kir6.2 K_ATP_ channels in HepG2C3A cells. Kinase inhibition and RNAi knockdown showed that the pinacidil activated K_ATP_ channels trigger signaling through Rho kinase and Janus kinase-3, and cause actin remodeling. The results are the first demonstration of a label-free methodology to characterize the composition and signaling of an endogenous ATP-sensitive potassium ion channel.

IIon channels are pore forming membrane proteins that coordinate diverse and vital functions including contraction, neurotransmission, secretion, and cell volume changes. Despite being implicated in a broad spectrum of diseases ranging from neurodegenerative disorders to diabetes, only 7% of all marketed drugs are targeted against ion channels^1^,^2^. Ion channels remain an underexploited target class in large part due to the lack of appropriate screening methods. Current technologies such as automated patch clamping, radioactive flux and ion-specific fluorescence dye assays are viable options but suffer from a common drawback – they only offer information about ion-channel opening/closing and potential upstream effectors^3^,^4^,^5^. As a result, our understanding of cellular pathways linked to ion channels remains poor relative to other membrane proteins such as G protein-coupled receptors (GPCRs) and receptor tyrosine kinases. This is exemplified by adenosine triphosphate (ATP) sensitive potassium (K_ATP_) channels. K_ATP_ channels serve as molecular sensors linking membrane excitability to metabolism^6^,^7^,^8^. The K_ATP_ channels are activated by interaction with intracellular Mg^2+^ADP and inhibited by high level of ATP, thus are sensitive to the energy state of cells^9^,^10^. K_ATP_ channels express in various tissues with different molecular compositions including cardiac myocytes, pancreatic β cells, smooth muscle cells, and neurons^6^,^11^. Cell plasma membrane K_ATP_ channel protein complex consists of four small pore-forming inward rectifying potassium channel subunits (Kir6.1 or Kir6.2) and four sulfonylurea receptors (SUR1, SUR2A, or SUR2B) as the regulatory β subunits^12^,^13^. Besides expression at the cell plasma membrane, the K_ATP_ channels are also found in the inner membrane of mitochondria^14^ and nuclear envelope^15^. Recent studies support that cardiac mitochondria K_ATP_ channels contain renal outer medullary potassium channel (ROMK), beside SUR2; however, their exact composition and organization remain elusive^16^,^17^,^18^. Advances in molecular biology and ion channel techniques have deepened our understanding about the assembly, expression, gating, structure, and regulation of K_ATP_ channels. However, little is known about cellular signaling mediated through K_ATP_ channels^9^,^10^,^11^,^12^,^13^,^19^.

Here we report the presence, composition and signaling of a functional K_ATP_ channel in HepG2C3A cells. This discovery was made possible by label-free cell phenotypic profiling with the aid of molecular and chemical biology tools. Label-free resonant waveguide grating (RWG) biosensor in microplate allows for non-invasive and real-time detection of cell phenotypic responses, termed dynamic mass redistribution (DMR), arising from the ligand-induced receptor activation in native cells^20^,^21^. The DMR signal obtained offers a holistic view of receptor signaling, so it is possible to deconvolute the systems cell biology^21^,^22^,^23^,^24^,^25^,^26^ and signaling waves^27^ of different classes of receptors. Taking advantages of the label-free biosensor to be highly sensitive and non-invasive, we used it to identify the DMR signature of pinacidil, a K_ATP_ channel opener^28^,^29^, across multiple cell lines, and deconvolute its origin and signaling pathways in HepG2C3A cells. We demonstrated that HepG2C3A cells express a functional K_ATP_ channel, although its location remains unknown.

## Results

### Label-free cell phenotypic profiling of ion channel ligands

**Figure 1:**
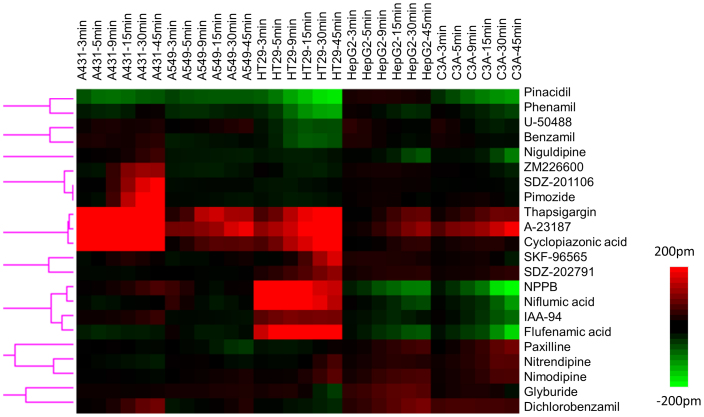
DMR heat map of ion channel ligands active in five cell lines, A431, A549, HT29, HepG2, and C3A. This heat map was obtained using clustering analysis of the DMR profiles of the ion channel ligands in the five cell lines. For each ligand, the real responses at six discrete time points post-stimulation (3, 5, 9, 15, 30, 45 min) in each cell line were used for the cluster analysis. Only ligands that led to a DMR greater than 50 pm, or less than −50 pm in at least one cell line were included in this analysis.

To identify functional receptors as well as appropriate cell line(s) for studying endogenous K_ATP_ channels, we first profiled a commercially available library consisting of seventy-two ion channel ligands, each at 10 μM, in the five distinct cell lines using DMR agonist assay in microplate^27^. The negative controls (that is, the cell response to the buffer solution containing 0.1% DMSO, equal amount to those for all ligands) were also included. Using the 3σ of the negative control response, we limited our analysis to active ligands that led to a DMR greater than 50 pm or smaller than −50 pm in at least one cell line. Results showed that out of seventy-two ligands twenty-two triggered robust DMR in at least one cell line (Fig. 1). Similarity analysis with the Ward hierarchical clustering algorithm and Euclidean distance metrics^26^ showed that these active ligands are divergent in their label-free cell phenotypic agonistic activity in these cell lines.

Detailed analysis revealed several interesting findings. First, thapsigargin, A23187, and cyclopiazonic acid shared similarity in their cell phenotypic agonistic activity. All three ligands triggered robust positive DMR in A431, A549, and HT29. However, only thapsigargin and A23187 were active in HepG2 and C3A cells. The non-selective agonistic activity of these ligands across the five cell lines, together with their similarity in DMR characteristics to those arising from the activation of G_q_-coupled receptors in respective cell lines^30^, suggests that their DMR is due to the increasing cytosolic calcium concentrations induced by these ligands. Thapsigargin is a non-competitive inhibitor of sarco/endoplasmic reticulum Ca^2+^ ATPase (SERCA)^31^, while cyclopiazonic acid is a specific inhibitor of SERCA^32^. Both inhibitors are known to raise cytosolic calcium concentration by blocking the ability of the cell to pump calcium into the sarcoplasmic and endoplasmic reticula, which leads to depletion of these stores and activates plasma membrane calcium channels, allowing an influx of calcium into the cytosol^31^. A23187 is a Ca^2+^ ionophore used extensively to mimic the effect of many physiological cell stimuli related to calcium ion^33^. The less robust response induced by cyclopiazonic acid is consistent with its relatively low potency, comparing to thapsigargin^32^. Due to its low potency, cyclopiazonic acid at 10 μM may not trigger the maximal response.

**Figure 2:**
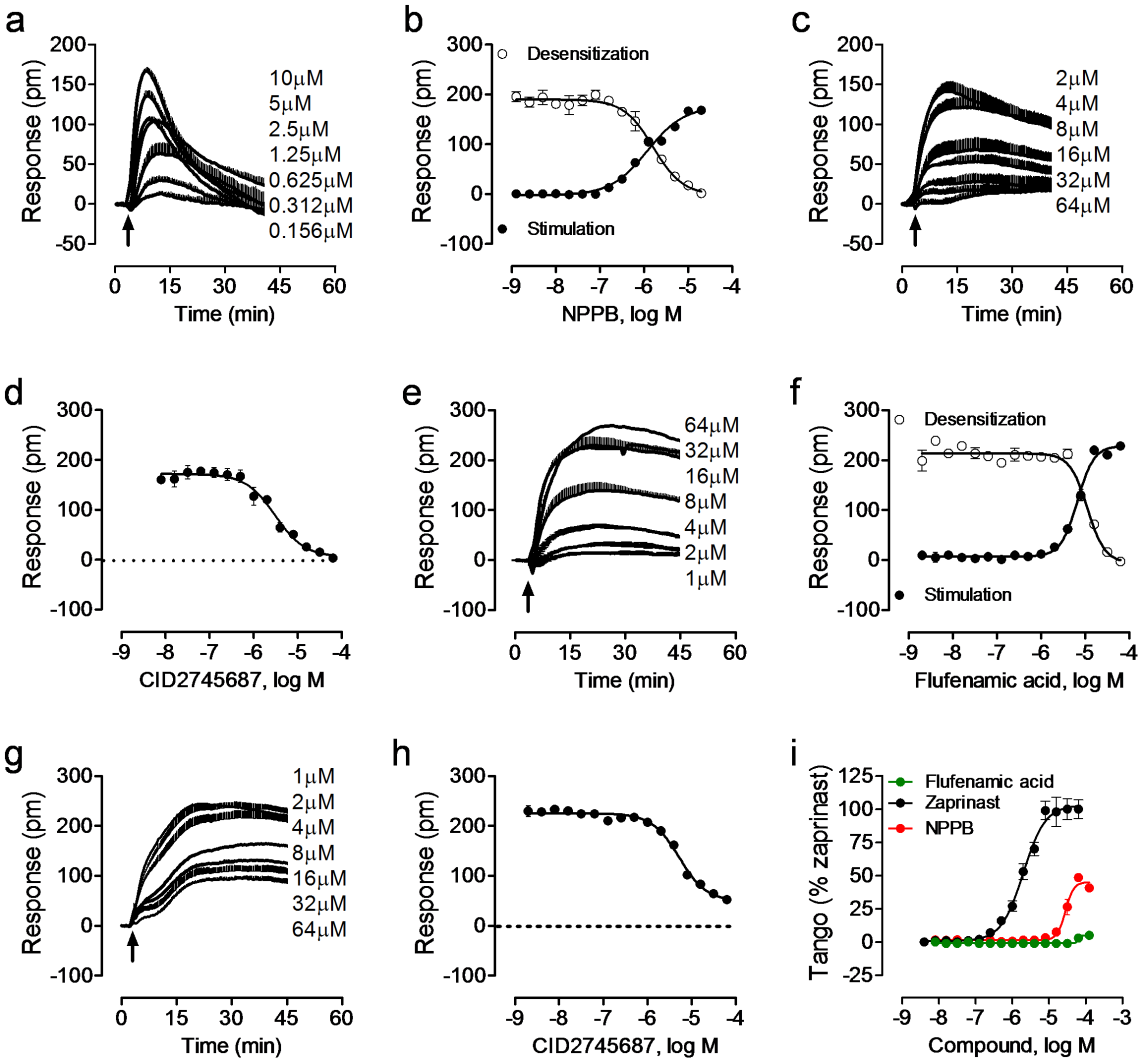
Agonist activity of two ion channel ligands, NPPB and flufenamic acid at the GPR35 in HT29 cells. (a) The real time DMR dose responses of NPPB. (b) The real DMR amplitudes of NPPB as a function of its dose, in comparison with the DMR of 1 μM zaprinast as a function of NPPB doses. (c) The real time DMR of 4 μM NPPB in the presence of CID2745687 at different doses. (d) The DMR amplitudes of 4 μM NPPB as a function of CID2745687 dose. (e) The real time DMR dose responses of flufenamic acid. (f) The DMR amplitudes of flufenamic acid as a function of its dose, in comparison with the DMR of 1 μM zaprinast as a function of flufenamic acid dose. (g) The real time DMR of 32 μM flufenamic acid in the presence of CID2745687 at different doses. (h) The DMR amplitudes of 32 μM flufenamic acid as a function of CID2745687 dose. (i) The β-arrestin Tango signal as a function of compound dose. For the desensitization, the cells were pretreated with respective ligands for 1 hr before stimulation with zaprinast at the fixed dose (1 μM). For the antagonist blockage, the cells were pretreated with CID2745687 at different doses for 1 hr before stimulation with respective agonist at a fixed dose. Data represents mean ± s.d. (n = 3).

Second, NPPB (5-nitro-2-(3-phenylpropylamino)benzoic acid), niflumic acid, IAA-94 (R(+)-methylindazone) and flufenamic acid shared similar cell phenotypic pharmacology; all were active in HT29 and C3A cells, and to less extent in HepG2 cells. Recently, we had showed that the positive DMR of niflumic acid in HT29 is due to the activation of GPR35^34^. Consistent with its known agonist activity at the GPR35^35^, NPPB triggered a dose-dependent DMR in HT29 (Fig. 2a), yielding an apparent logEC_50_ of −5.95 ± 0.07 (n = 3) (Fig. 2b). It also desensitized the response to 1 μM zaprinast, a known GPR35 agonist, with a logIC_50_ of −5.79 ± 0.05 (n = 3) (Fig. 2b). The known GPR35 antagonist CID2745687 dose-dependently and completely blocked the DMR of 4 μM NPPB (n = 3) (Fig. 2c), with an apparent logIC_50_ of −5.51 ± 0.06 (n = 3) (Fig. 2d). Although flufenamic acid was reported to be inactive in human GPR35-β-arrestin-2 interaction assays^36^, we found that flufenamic acid is a DMR biased partial agonist at the GPR35 (Fig. 2e–h). Flufenamic acid triggered a dose-dependent DMR in HT29 (Fig. 2e), yielding an apparent logEC_50_ of −5.18 ± 0.03 (n = 3) (Fig. 2f). It also desensitized the cells responding to the second stimulation with 1 μM zaprinast with a logIC_50_ of −4.95 ± 0.06 (n = 3) (Fig. 2f). CID274568 dose-dependently and partially blocked the DMR of 32 μM flufenamic acid with a logIC_50_ of −5.28 ± 0.05 (Fig. 2g & h). Using GPR35 Tango β-arrestin gene reporter assay, we found that NPPB triggered a dose response with a logEC_50_ of −4.55 ± 0.04 and a maximal response that is 45 ± 3% (n = 3) of the full agonist zaprinast, but flufenamic acid was inactive (Fig. 2i). These results suggest that similar to several other GPR35 agonists including benserazide^37^, tolcapone^38^ and rosmarinic acid^39^, flufenamic acid not only activates the endogenous GPR35 in HT29, but also activates an additional unknown receptor. Of note, we did not determine the mechanism accounted for the DMR of IAA-94 in HT29, given that the IAA-94 DMR is much smaller than those induced by GPR35 agonists.

Third, pimozide, SDZ-201106 and ZM226600 all triggered characteristically similar DMR specifically in A431. ZM226600 is a Kir6 K_ATP_ channel opener with an EC_50_ of 0.5 μM^40^. Pimozide is a dopamine D_2_ antagonist and has high affinity to 5-HT_7_ receptor^41^. SDZ-201106 is a sodium channel opener^42^. Given that no common mode of action is known among these ligands, it is possible that they activate an unknown receptor in A431 cells; this possibility is currently under investigation.

Fourth, pinacidil was found to trigger a robust negative DMR in A431, A549, HT29 and C3A, but not in HepG2 cells. However, several other K_ATP_ openers including diazoxide, minoxidil, minoxidil sulphate, and PCO-400 in the library, each at 10 μM, were inactive in the DMR agonist profiling across the five cell lines. Given that K_ATP_ openers are classified based on their ability to open the channel *per se*, but the DMR of a ligand reflects the functional consequence of receptor-ligand interaction in cells, we postulated that similar to GPCR ligands^43^, K_ATP_ openers may also display functional selectivity and the DMR of pinacidil is related to the signaling of endogenous K_ATP_ channels. Of note, it was reported that C3A expresses K_ATP_ channels that play a role in cell proliferation^44^, and HepG2 has little K_ATP_ channel current on the cell surface but the K_ATP_ channel expression can be induced by transfection of insulin and glucose transporter GLUT2^45^.

Together, these analyses suggest that the label-free cell phenotypic profiling approach is powerful for determining the on-target and off-target activity of ion channel ligands. Given the unique signature of pinacidil, we herein were primarily focused on the deconvolution of the target and pathways activated by pinacidil.

### The pinacidil DMR in C3A cells is due to the activation of endogenous K_ATP_ channels

**Figure 3:**
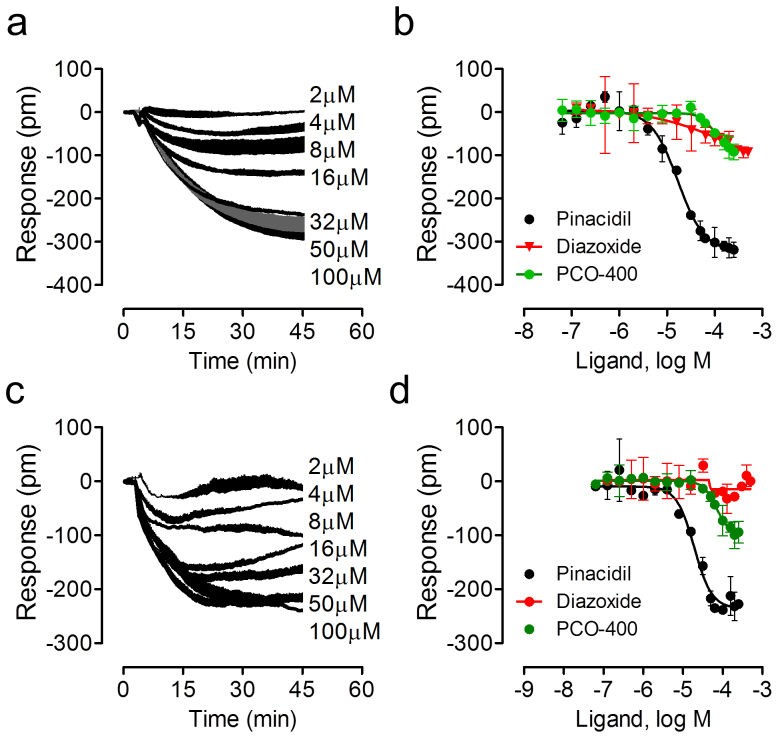
Dose responses of K_ATP_ openers in C3A and A549 cells. (a) The real-time DMR dose responses of pinacidil in C3A cells. (b) The real DMR amplitudes at 50 min post stimulation as a function of ligand dose in C3A cells. (c) The real-time DMR dose responses of pinacidil in A549 cells. (b) The real DMR amplitudes at 50 min post stimulation as a function of ligand dose in A549 cells. Data represents mean ± s.d. (n = 6 for pinacidil, and n = 3 for other openers).

To determine the composition of endogenous K_ATP_ channels, we first characterized the dose responses of known K_ATP_ openers using DMR agonist assay. Results showed that pinacidil triggered a dose response in C3A cells (Fig. 3a), yielding a logEC_50_ of −4.77 ± 0.05 (n = 6) (Fig. 3b). In comparison, both PCO-400 and diazoxide triggered much smaller negative DMR with lower potency; their logEC_50_s were −3.99 ± 0.07 and −3.47 ± 0.09 (n = 3), respectively (Fig. 3b). In contrast, minoxidil up to 64 μM was inactive. Similarly, pinacidil also triggered a dose response in A549 cells (Fig. 3c), yielding a logEC_50_ of −4.72 ± 0.05 (n = 6) (Fig. 3d). In comparison, PCO-400 triggered a much smaller negative DMR with a logEC_50_ of −4.16 ± 0.03 (n = 3), while both diazoxide and monoxidil were inactive (Fig. 3d; data not shown). The relatively low potency of active K_ATP_ openers obtained may be related to the whole cell measurements, wherein the intracellular ATP/ADP ratio has direct effects. Another possibility is that the K_ATP_ channels are located inside cells, and the effective intracellular concentration of these openers is a function of cell uptake and efflux and may be lower than those added extracellularly. Of note, in order to better visualize the DMR characteristics we used real DMR responses, instead of their absolute values, for all dose response analysis. Nonetheless, these results suggest that these openers display distinct potency and efficacy to trigger DMR in C3A and A549 cells.

**Figure 4:**
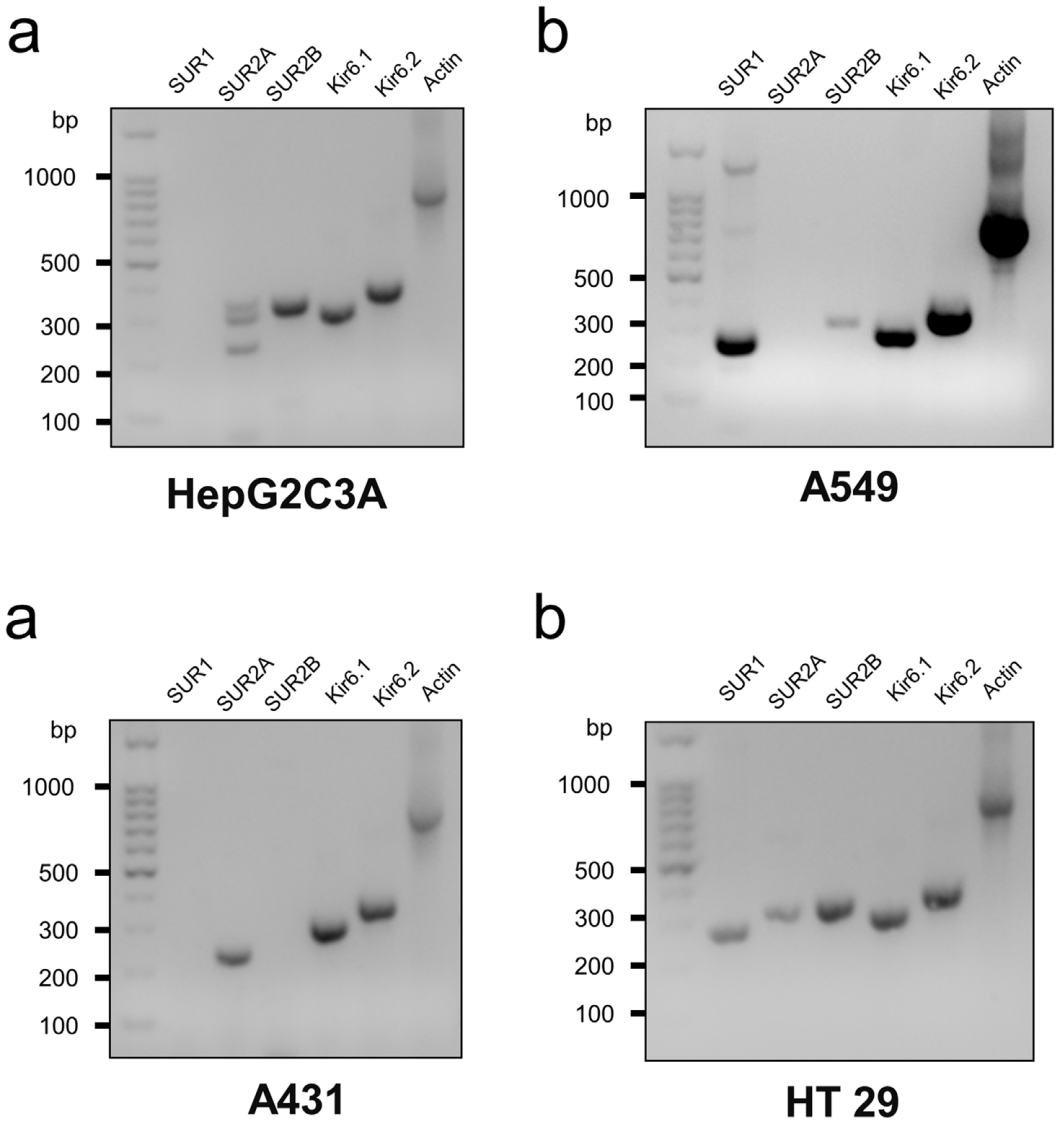
mRNA expression patterns of K_ATP_ channels in different cell lines. (a) C3A; (b) A549; (c) A431; (d) HT29 cells.

Second, we performed reverse transcriptase PCR to determine the mRNA expression of K_ATP_ channels. Results showed that C3A endogenously expresses mRNAs for SUR2B, Kir6.1, and Kir6.2, to less extent SUR2A, but not SUR1 (Fig. 4a). A549 expresses mRNAs for SUR1, Kir6.1 and Kir6.2, but not SUR2A and SUR2B (Fig. 4b). A431 expresses mRNAs for SUR2A, Kir6.1 and Kir6.2, but not SUR1 and SUR2B (Fig. 4c). HT29 expresses mRNAs for primarily SUR2B, Kir6.1 and Kir6.2, and to less extent SUR1 and SUR2A (Fig. 4d). These results suggest that different cell lines have different expression patterns of K_ATP_ channels.

**Figure 5:**
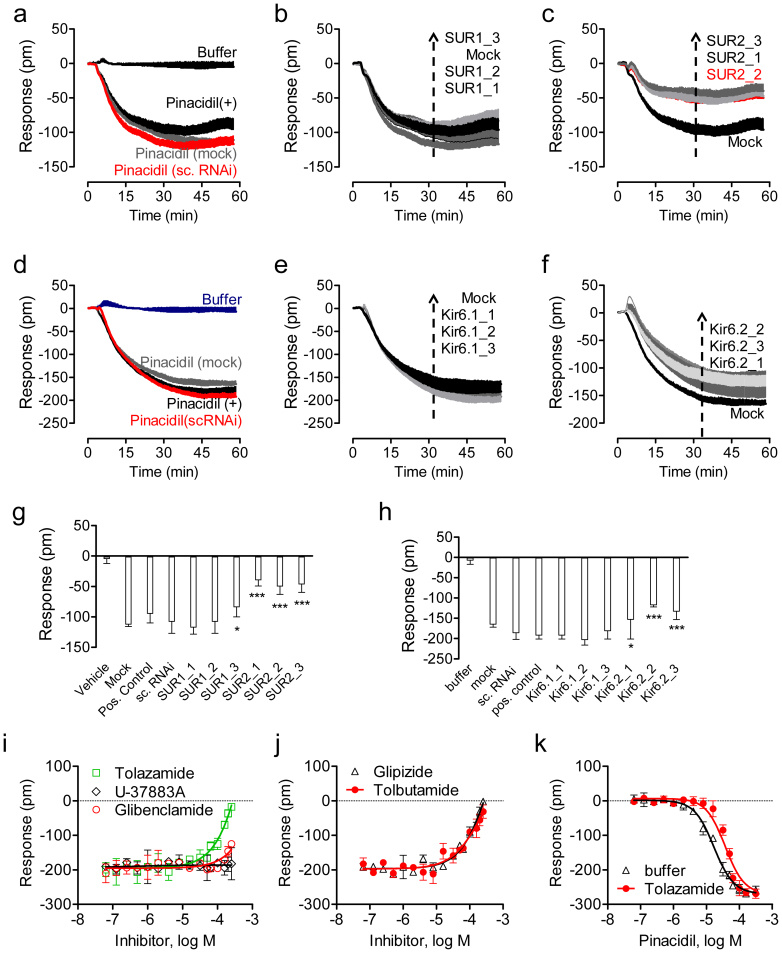
The RNAi knockdown effect on the DMR of 40 μM pinacidil in C3A cells. (a,d) The DMR of pinacidil in untreated, mock transfection treated (mock) and scrambled RNAi (sc. RNAi) treated C3A cells, in comparison with the negative control. (b) The DMR of pinacidil in the mock transfection and three SUR1 RNAi treated cells. (c) The DMR of pinacidil in the mock transfection and three SUR2 RNAi treated cells. (e) The DMR of pinacidil in the mock transfection and three Kir6.1 RNAi treated cells. (f) The DMR of pinacidil in the mock transfection and three Kir6.2 RNAi treated cells. (g,h) statistical analysis of the effects of RNAi knockdown on the pinacidil DMR. *, *p* < 0.05; ***, *p* < 0.001. (i,j) The DMR of 32 μM pinacidil in C3A cells as a function of K_ATP_ blockers. The cells were first treated with each blocker at different doses for 1 hr, followed by stimulation with 32 μM pinacidil. (k) The impact of co-existence of 40 μM tolazamide on the potency of pinacidil. For (g–k), the pinacidil DMR amplitudes at 50 min post stimulation were plotted. Data represents mean ± s.d. (n = 6 for a–h; n = 3 for i–k).

Third, we applied RNAi knockdown to determine the functional K_ATP_ channels in C3A cells. Results showed that the mock or scrambled RNAi transfection had little impact on the pinacidil DMR (Fig. 5a). The treatment with three RNAi against SUR1 had little impact on the pinacidil DMR (Fig. 5b), consistent with the absence of SUR1 mRNA in C3A cells. However, knockdown of SUR2 with three RNAi all markedly suppressed the pinacidil DMR (Fig. 5c). Similar to the mock or scrambled RNAi transfection (Fig. 5d), three RNAi knockdown of Kir6.1 had little impact on the pinacidil DMR (Fig. 5e). In contrast, the knockdown of Kir6.2 with three RNAi all markedly suppressed the pinacidil DMR (Fig. 5f). One-way ANOVA analysis suggests that RNAi for SUR2 and Kir6.2, but neither SUR1 or Kir6.1, significantly altered the pinacidil DMR (Fig. 5g and h). Given the low expression level of these proteins and the moderate efficiency of typical RNAi knockdown in our laboratory^26^ (also see below), we didnot attempt to use Western blot to quantify the knockdown efficiency of respective RNAi.

Fourth, the effects of different K_ATP_ inhibitors on the pinacidil DMR were examined and used to further strengthen RNAi knockdown results. Results showed that three sulfonylurea blockers including tolazamide, glipizide and tolbutamine all dose-dependently blocked the pinacidil DMR, leading to a single logIC_50_ of −3.85 ± 0.09, 3.94 ± 0.10, and 3.91 ± 0.10 (n = 3), respectively. In contrast, glibenclamide up to 250 μM only partially attenuated the pinacidil DMR, and U-37883A had little impact on the pinacidil DMR (Fig. 5i & j). U-37883A is a non-sulfonylurea blocker that has been reported to selectively inhibit Kir6.1 containing K_ATP_ channels^46^, while glibenclamide is a relatively selective SUR1 inhibitor^47^,^48^. Furthermore, the co-existence of 40 μM tolazamide caused the right shift of pinacidil's potency (Fig. 5k). Together, these results suggest that the pinacidil DMR in C3A is due to the activation of SUR2/Kir6.2 K_ATP_ channels. Of note, we attempted, but failed to conclusively determine the exact location of K_ATP_ channels in C3A cells, due to very low levels of K_ATP_ channel proteins.

Collectively, these pharmacological profiling, RNAi knockdown, and RT-PCR results suggest that C3A endogenously expresses a functional SUR2/Kir6.2 K_ATP_ channel, whose activation by pinacidil results in a robust DMR signal.

### Kinase inhibition profiling

**Figure 6:**
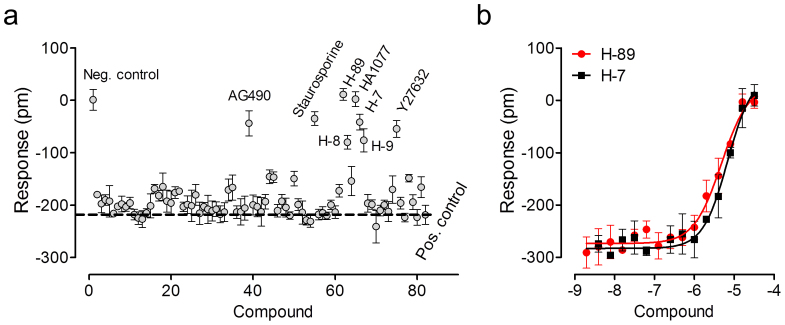
The sensitivity of the pinacidil DMR in C3A cells to kinase inhibition. (a) The 32 μM pinacidil DMR amplitudes at 50 min post stimulation were plotted as a function of compound. The pinacidil DMR was obtained after pretreatment with inhibitors, all at 10 μM, for 1 hr. (b) The dose-dependent inhibition of the 32 μM pinacidil DMR amplitudes at 50 min post stimulation by H-89 and H-7. Data represents mean ± s.d. (n = 4 for all).

The regulation of K_ATP_ channels has been extensively investigated; in particular, its upstream regulation mediated by protein kinase A (PKA) has been well documented^49^,^50^,^51^,^52^. The phosphorylation of ion channels by protein kinases is an important mechanism by which membrane excitability is regulated by signaling pathways. However, a clear understanding about the signaling of K_ATP_ is still lacking. Thus, we investigated the impact of a library of known kinase inhibitors on the pinacidil DMR in C3A cells. Results showed that the majority of kinase inhibitors had little impact on the pinacidil DMR (Fig. 6a). However, a subset of inhibitors markedly suppressed the DMR of pinacidil. Among them, H-89, HA-1077, H-7, and H-8 are known PKA inhibitors, while AG490 is a putative Janus activated kinase (JAK) inhibitor, Y27632 is a ROCK inhibitor, and the alkaloid staurosporine is a broad spectrum, high affinity kinase inhibitor with highest affinity for protein kinase C (0.7 nM). DMR inhibition assay showed that H-89 and H-7 dose-dependently and completely blocked the DMR of 32 μM pinacidil, leading to a logIC_50_ of −5.32 ± 0.09 and −5.17 ± 0.10, respectively (Fig. 6b). The sensitivity of the pinacidil DMR to these PKA inhibitors were consistent with the previous findings, which suggest that PKA is anchored in proximity to K_ATP_ channels in the caveolae^49^, and more than one site in Kir6.2 (e.g., S372 or T224 in Kir6.2)^51^ and SUR (e.g., T633 and S1465 in SUR2B)^49^ have been implicated in PKA phosphorylation. The positive regulation of K_ATP_ by PKA was evidenced by the observed attenuation of K_ATP_ channel currents in the presence of PKA inhibitors^49^,^50^,^51^,^52^. Of note, compounds containing 5-isoquinolinsulfonyl moieties such as H-89 also bind directly to the SUR subunit but with relatively low potency^53^. At the concentration range tested it is not possible to distinguish between direct blocking of the K_ATP_ channel and indirect inhibition through abrogation of PKA activity. The inhibition of the pinacidil DMR by staurosporine may also be linked to PKA (Ki ~ 7 nM), but the prevalence of other mechanisms cannot be ruled out^54^,^55^. Nonetheless, these results suggest that the pinacidil DMR can be modulated by kinase activity.

### JAK3 and JAK2 are involved in K_ATP_ channel signaling

**Figure 7:**
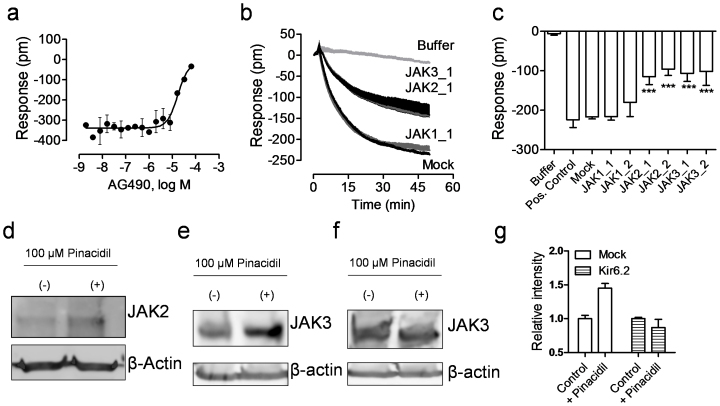
JAK2 and JAK3 were involved in the K_ATP_ signaling in C3A cells. (a) The DMR amplitudes of 40 μM pinacidil at 50 min post stimulation as a function of AG490 dose. The cells were treated with AG490 for 1 hr, followed by pinacidil stimulation. (b) The real-time DMR of 40 μM pinacidil in C3A cells with mock transfection (mock) or JAK siRNA. The buffer DMR was included as a negative control. (c) The pinacidil DMR amplitudes at 50 min post stimulation as a function of treatment. (d) Western blot using anti-JAK2 for cell lysate without (−) and with (+) 100 μM pinacidil treatment. (e) Western blot using anti-JAK3 for the lysate of mock transfected cells without (−) and with (+) 100 μM pinacidil treatment. (f) Western blot using anti-JAK3 for the lysate of the Kir6.2 siRNA treated cells without (−) and with (+) 100 μM pinacidil treatment. (g) The relative intensity of JAK3 protein in the mock transfected or the Kir6.2 siRNA treated cells without (Control) and with 100 μM pinacidil treatment (+Pinacidil). Data represents mean ± s.d. (n = 6 for a–c; n = 3 for g).

Janus kinases (JAK1, 2, 3) are protein tyrosine kinases involved in cytokine mediated cellular signaling and are crucial for a variety of cellular functions including cellular survival, proliferation, differentiation and apoptosis^56^,^57^. Given the attenuation of the pinacidil DMR by AG490, we examined the role of JAKs in K_ATP_ channel signaling using multiple assays. First, DMR inhibition assay showed that AG490 dose-dependently suppressed the pinacidil DMR, yielding a logIC_50_ of −4.77 ± 0.10 (n = 3) (Fig. 7a). Second, siRNA knockdown studies showed that the treatment with two siRNAs for JAK1 had little impact on the pinacidil DMR, but two siRNAs for JAK2 and two for JAK3 all markedly suppressed the pinacidil DMR (Fig. 7b and c). Third, western blotting showed that C3A cells primarily express JAK3, to less extend JAK2, but not JAK1. Moreover, the treatment of cells with 100 μM pinacidil markedly increased the level of JAK2 (Fig. 7d) and JAK3 (Fig. 7e). Importantly, the Kir6.2 siRNA treatment impaired the pinacidil induced increase of JAK3 protein level (Fig. 7f and g), suggesting that the pinacidil induced increase in JAK3 protein is due to the activation of Kir6.2 K_ATP_. We also attempted, but failed, to detect p-JAK levels in the absence and presence of pinacidil. Several possible mechanisms are accounted for this; one may be due to the poor activity of anti-p-JAK antibodies used; another is that the pinacidil activated K_ATP_ did not cause phosphorylation of JAKs, but only increased the expression level of JAKs. Of note, we used moderately high dose (40 μM) of pinacidil for DMR profiling of the effect of kinase inhibitor and RNAi knockdown in order to improve sensitivity, but a higher dose (100 μM) for JAK expression analysis to achieve maximal effect. Nonetheless, these results suggest the important role of JAK3, to less extent JAK2, in the K_ATP_ channel signaling in C3A cells.

### K_ATP_ channel signaling is linked to ROCK and actin remodeling

**Figure 8:**
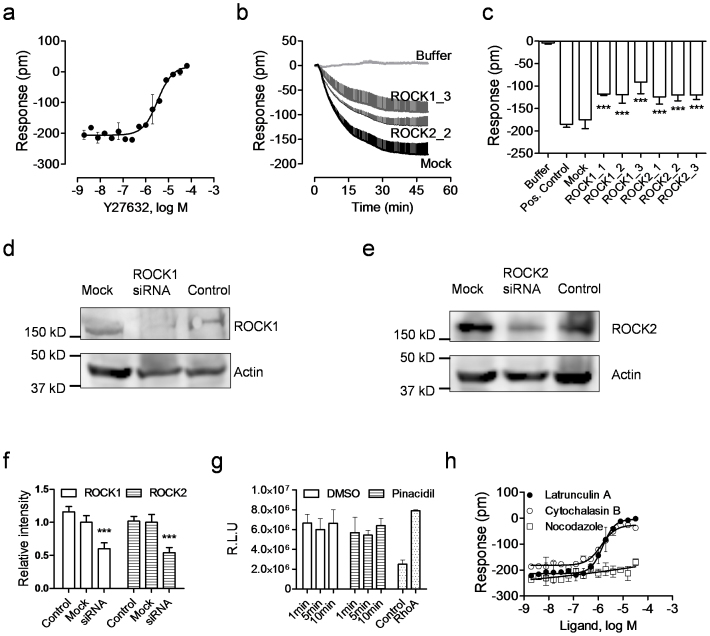
ROCK is involved in the K_ATP_ signaling in C3A cells. (a) The DMR amplitudes of 40 μM pinacidil at 50 min post stimulation as a function of Y27632 dose. The cells were treated with Y27632 for 1 hr, followed by pinacidil stimulation. (b) The real-time DMR of 40 μM pinacidil in C3A cells with mock transfection (mock) or ROCK siRNA. The buffer DMR was included as a negative control. (c) The pinacidil DMR amplitudes at 50 min post stimulation as a function of treatment. (d) Western blot using anti-ROCK1 for cell lysate of the untransfected, mock transfected, or ROCK1 siRNA treated cells. (e) Western blot using anti-ROCK2 for cell lysate of the untransfected, mock transfected, or ROCK1 siRNA treated cells. (f) The relative intensity of ROCK1/ROCK2 protein in the untransfected, mock transfected, or ROCK1 siRNA treated cells. (g) The ELISA responses of RhoA activity. The cells were treated with 100 μM pinacidil for the specified time, or equal amount DMSO. The blank control was also compared with the supplied RhoA protein to determine the background. (h) The effect of microfilament inhibitors on the pinacidil DMR in C3A cells. The pinacidil DMR amplitudes at 50 min post stimulation were plotted as a function of three inhibitors, which were used to pretreat the cells. Data represents mean ± s.d. (n = 6 for a–c; n = 3 for f–h).

Rho kinases (ROCK1 and ROCK2) play important roles in the small GTPase RhoA initiated signaling pathways. Rho kinases are known to be involved in a variety of cellular functions including cytoskeleton organization, cell proliferation and apoptosis^58^. Given the ability of Y27632 to attenuate the pinacidil response, we next examined the role of ROCK in the K_ATP_ signaling in C3A cells. DMR inhibition assay showed that Y27632 dose-dependently inhibited the pinacidil DMR, yielding a logIC_50_ of −5.47 ± 0.07 (n = 3) (Fig. 8a). RNAi knockdown of ROCK1 or ROCK2 was found to markedly attenuate the pinacidil DMR (Fig. 8b and c). Western blotting confirmed that the efficiency of ROCK RNAi knockdown was about 40–50% (Fig. 8d–f), consistent with the level of suppression of the pinacidil DMR by these siRNAs (comparing Fig. 8f with Fig. 8c). RhoA activity results showed that the treatment of C3A cells with pinacidil had little impact on the RhoA activity (Fig. 8g), suggesting that the pinacidil activated K_ATP_ channels had no effect on the activity of RhoA, the upstream effector of ROCK.

Given the important roles of ROCK signaling in actin remodeling, we finally examined the impact of microfilament modulators on the pinacidil response in C3A cells. Results showed that both cytochalasin B and latrunculin A dose-dependently blocked the pinacidil DMR, leading to a logIC_50_ of −5.87 ± 0.03 and −5.81 ± 0.04 (n = 3) (Fig. 8h). Cytochalasin B acts by capping growing actin microfilaments, while latrunculin A acts by sequestering actin monomers (Ki ~ 0.2–0.4 μM). In contrast, the microtubule disrupting agent nocodazole up to 32 μM had little effect on the pinacidil DMR (Fig. 8h). These results suggest that the K_ATP_ channel initiated signaling in liver cells is linked to ROCK activity, leading to actin remodeling.

## Discussion

We here present a strategy centered on label-free cell phenotypic profiling to discover the composition and signaling of an endogenous K_ATP_ channel in C3A cells. Label-free cell phenotypic profiling of a compound library consisting of seventy-two ion channel ligands across the five different cell lines led to identification of twenty-two ligands that displayed agonistic activity in at least one cell lines. Several classes of active ligands can be assigned based on their known mechanism of action (e.g., Ca^2+^ mobilizing agents such as A23187, thapsigargin and cyclopiazonic acid), or using orthogonal assays (e.g., GPR35 agonists flufenamic acid, niflumic acid and NPPB).

Combining DMR RNAi knockdown, ligand pharmacology profiling, and RT-PCR has allowed us to ascertain the presence, composition and signaling of a functional SUR2/Kir6.2 K_ATP_ ion channel in C3A cells. K_ATP_ openers are a structurally diverse group of compounds, and also diverse in the modes of action. Diazoxide is a benzothiadiazine dioxide, pinacidil a cyanoguanidine, PCO-400 a benzopran, minoxidil a pyrimidine N-oxide sulfate, and ZM226600 an anilide tertiary carbinol. The Kir6.2/SUR1 channel can be activated by diazoxide but is relatively insensitive to pinacidil, while the Kir6.2/SUR2A channel is only weakly responsive to diazoxide but can be activated by pinacidil^59^. On the other hand, the Kir6.2/SUR2B channel can be activated by diazoxide^60^; ZM226600 can activate a channel consisting of SUR2B co-associated with Kir6.1 or Kir6.2^61^; minoxidil can specifically activate Kir6.1/SUR2 channels^62^. Our DMR profiling showed that among all KCOs tested in C3A cells pinacidil triggered a robust DMR, while PCO-400 and diazoxide are less active, ZM226600 and minoxidil were inactive. Considering gene expression, RNAi knockdown and K_ATP_ inhibitor profiling data, our DMR results are better explained by the presence of a functional Kir6.2/SUR2A channel in C3A cells. Of note, due to the low expression of K_ATP_ channels, in particular Kir6.2 subunits at both mRNA and protein levels in C3A cells, we have difficulty to determine the exact location of these channels whose activation contributes to the DMR of pinacidil. We also did not determine the location and compositions of K_ATP_ channels in other pinacidil responsive cell lines. Nonetheless, these results demonstrate the use of label-free cell phenotypic profiling for broad pharmacological profiling of K_ATP_ channels.

The molecular events underlying the modulation of K_ATP_ activity by protein kinases is poorly understood. Both JAK2 and ROCK inhibitors have been reported to be physiologically linked to K_ATP_ channels in cardioprotection. The JAK/STAT pathway mediates opioid induced cardioprotection via glycogen synthase kinase-3β; AG490 abrogates this cardioprotective effect^63^. The proposed mechanism for reduced myocardial no-reflow during ischemic preconditioning, a strategy also used for reperfusion therapy following acute myocardial infarction, involves the opening of K_ATP_ channels via inhibition of ROCKs^64^. ROCK inhibitors decrease the area of no-reflow; K_ATP_ inhibitors abolish the reduced no-reflow. Although the exact cellular pathways undetermined, we here showed that both JAK3 (to less extent JAK2) and ROCK are involved in the K_ATP_ channel signaling in C3A cells, a liver cell line.

Information regarding the role and pharmacology of K_ATP_ channels in hepatocytes is limited. While the physiological outcomes of K_ATP_ channel activity in hepatocytes and cardiomyocytes are expected to be different, it is likely that their modulation by kinases occur through similar molecular pathways. The ability to capture complex physiological phenomena surrounding ion channel activity in experimentally convenient cellular screening assays, as described herein, is powerful and unprecedented. Label-free DMR assays offer great flexibility in assay formats^27^, rich information content with real-time kinetics, high throughput, and more importantly, holistic view of receptor signaling and drug pharmacology with wide coverage of pathway/targets^65^, including ion channels as demonstrated here. DMR assays not only permit pharmacological profiling of distinct types of ligands (openers and inhibitors) for the channel proteins, but also allow for investigating the signaling mediated through ion channels, later of which is difficult to be assessed using electrophysiology. However, DMR assays also come with several limitations. First, the label-free assays cannot detect directly the ion flux related to the open and close of ion channels. Second, the pharmacological profiles of ligands for a specific ion channel may not correlate with those obtained using electrophysiology, given that the DMR is a whole cell phenotypic response linking to the functional responses of cells upon the activation of ion channels. Third, without validation using conventional approaches, label-free is difficult to differentiate the upstream (e.g., PKA phosphorylation of the K_ATP_ channels), and downstream (e.g., the K_ATP_ activation increased JAK expression) signaling events of the channels. Thus, label-free biosensor enabled cell phenotypic profiling for investigating ion channel pharmacology is an attractive complimentary technology to electrophysiology and other conventional techniques.

## Methods

### Materials

BioMol kinase inhibitor library and latrunculin A was obtained from Enzo Life Sciences, Inc. (Farmingdale, NY, USA). CID2745687, diazoxide, flufenamic acid, minoxidil, and pinacidil were obtained from Tocris Chemical Co. (St. Louis, MO, USA). PCO-400 was obtained from Santa Cruz Biotech Inc. (Santa Cruz, CA, USA). AG490, cytochalasin B, glibenclamide, glipizide, tolazamide, tolbutamide, U-27883A, and Y27632 were purchased from Sigma-Aldrich (St. Louis, MO, USA). Cell culture reagents were purchased from Life Technologies (Grand Island, NY, USA). The kinase inhibitor library, each at 10 mM in dimethyl sulfoxide (DMSO), were freshly diluted using the assay buffer (HBSS; 1× Hanks' balanced salt buffer, 10 mM Hepes-KOH, pH 7.1) before use. All other compounds were dissolved in DMSO at 100 mM and stored at −80°C. Epic® 384-well biosensor cell culture compatible microplates were obtained from Corning Incorporated (Corning, NY, USA).

### Cell culture

All five cell lines were obtained from American Type Cell Culture (ATCC) (Manassas, VA, USA). They are human epidermoid carcinoma A431, adenocarcinomic human alveolar basal epithelial cell A549, human colorectal adenocarcinoma HT-29, human hepatocellular carcinoma HepG2 and its clonal derivative C3A (HepG2C3A). C3A is a clonal derivative of HepG2 that was selected for strong contact inhibition of growth, high albumin production, high production of alpha fetoprotein and ability to grow in glucose deficient medium. All cells were subcultured 1–2 times per week according to ATCC's instruction. Cell passage less than 15 was used for all experiments. All cells were passaged at 37°C with 5% CO_2_ using the following complete medium: McCoy's 5A medium for HT-29, Eagle's Minimum Essential Medium (MEM) for HepG2 and HepG2C3A, F-12K Medium for A549, and Dulbecco's Modified Eagle's Medium (DMEM) for A431. All media were supplemented with 10% fatal bovine serum, 4.5 g/L glucose, 2 mM glutamine, 100 μg/ml penicillin and streptomycin. All cells were passed with trypsin/ethylene-diaminetetraacetic acid when approaching 90% confluence to provide new maintenance culture on T-75 flasks and experimental culture on the biosensor microplates.

For DMR assays, the cell seeding density was optimized for each cell line. The optimal seeding density was found to be 32 K, 25 K, 25 K, 21 K, and 21 K per well for HT-29, A431, A549, HepG2 and C3A cells, respectively. After overnight culture (~20 hrs), all cells were directly washed using the HBSS buffer before assay, except for A431 which was subject to starvation for an extra day using the serum-free DMEM medium. The cell confluency was ~95% at the time of assaying for all cells.

### Reverse transcriptase PCR

Total RNA was extracted from one T-75 flask with a confluent monolayer of cells (15–30 million cells) for each cell line using an RNeasy mini kit (Qiagen, Cat#74104, Valencia, CA). On-column DNase digestion was performed using RNase-free DNase set (Qiagen, Cat#79254) to eliminate genomic DNA contamination. The concentration and quality of total RNA were determined using a Nanodrop 8000 (Thermo Scientific). The primer sequences used were based on previous reports^66^,^67^ and custom synthesized through Sigma-Aldrich. Reverse transcriptase PCR was performed using One-Step RT-PCR kit from Qiagen Inc. (Cat# 210212). The PCR conditions were as follows: 50°C for 30 min, 95°C for 15 min, followed by 60 cycles of 1 min at 94°C, 1.5 min at 57°C and 2 min at 72°C, with a final extension of 10 min at 72°C. For all gel electrophoresis analysis equal amount of DNA was loaded. The DNA of actin obtained was used the control. The comparative level was estimated across different genes in a specific cell line, not across different cell lines.

### RNAi knockdown

All siRNAs including universal scrambled RNAi were selected from the pre-designed siRNA database of Sigma-Aldrich. For each target the top two or three siRNA having predicted high knockdown efficiency was selected. The siRNA IDs are SASI_Hs01_00092347, SASI_Hs01_00092348 and SASI_Hs01_00092349 for SUR1 (Refseq ID: NM_000352); SASI_Hs01_00061301, SASI_Hs01_00061302 and SASI_Hs01_ 00061303 for SUR2 (Refseq ID: NM_005691); SASI_Hs01_ 00113357, SASI_Hs01_00113358 and SASI_Hs01_00113359 for Kir6.1 (Refseq ID: NM004982); SASI_Hs01_00220256, SASI_ Hs01_00220257 and SASI_Hs01_00220258 for Kir6.2 (Refseq ID: NM000525); SASI_Hs01_00065573, SASI_Hs01_00065571, and SASI_Hs01_00065570 for ROCK1 (RefseqID: NM_005406); SASI_Hs01_00204253, SASI_Hs01_00204252, and SASI_Hs01_00204251 for ROCK2 (RefseqID: NM_004850); SASI_Hs01_ 00174614 and SASI_Hs01_ 00174613 for JAK1 (ReqID: NM_002227). SASI_Hs02_ 00338675 and SASI_Hs01_00041547 for JAK2 (ReqID: NM_004972); SASI_Hs01_00118128 and SASI_Hs02_ 00302103 for JAK3 (ReqID: NM_000215).

siRNA transfection was performed using the N-TER nanoparticle siRNA Transfection system (Cat# N2913) from Sigma. Specifically, 5000 cells were first plated into each well of an Epic® 384well microplate, and cultured for 20 hrs using the complete medium. The cells were then transfected with 50 nM siRNA and incubated in siRNA containing medium for 24 hrs before replaced with fresh cell culture medium. Label-free DMR assays were performed 48 hrs after transfection. The cells treated with the transfection vehicle were used as the mock control.

### Immunoprecipitation and Western blotting

C3A cells were plated in 6-well tissue culture treated plate with 3 × 10^5^ cells per well. Cells were transfected with respective top-rank siRNA at 50 nM final concentration 20 hrs after plating. 24 hrs after transfection the transfection reagent containing media were removed and replaced with the respective complete media. 48 hrs after transfection cells were lysed using 1% NP40 lysis buffer (150 mM NaCl, 25 mM Tris-HCl, pH 7.6, 1% NP-40) with the protease inhibitor cocktail (Roche Applied Science, Indianapolis, IN, USA). The cell lysate was then centrifuged at 14,000 rpm for 20 min. The supernatant was then transferred to a new tube before western blotting.

For west blotting, 120 μl cell lysate of each sample were mixed with 40 μl 4× SDS sample buffer (Bio-Rad Life Sciences, Hercules, CA, USA), and then boiled at 100°C for 5 min. Proteins were separated on 10% SDS gel, and 15 μl of each sample was loaded to the gel. For ROCK1 and 2, membrane was blotted with either rabbit anti-ROCK1 (Santa Cruz Biotech, catalog # sc-5560), or rabbit anti-ROCK2 (sc-5561), or goat anti-Actin (sc-1616) (1:500 dilution) for 1 hr, and then with 2nd HRP conjugated goat anti-rabbit or horse anti-goat antibody (1:2000 dilution) for 15 min. Similarly, for JAKs, cell lysate was immunoprecipitated with rabbit anti-JAK1 (sc-7228), goat anti-p-JAK1 (Tyr 1022/Tyr 1023) (sc-16773), rabbit anti-JAK2 (sc-294), goat anti-p-JAK2 (Tyr 1007/Tyr 1008) (sc-21870), rabbit anti-JAK3 (sc-513) or goat anti-p-JAK3 (Tyr 980) (sc-16567) (1:250) at 4°C and then with 2nd HRP conjugated goat anti-rabbit or horse anti-goat antibody (1:2000 dilution). The images were developed with ECL-Plus (GE Healthcare). The original west blot images were presented in Supplementary Figures S1 to S5.

### DMR assays

DMR assays in microplate were performed using high throughput screening compatible Epic® system (Corning)^68^. This system consists of a temperature-control unit (26°C), an optical detection unit, and an on-board liquid handling unit operated by robotics. This system scans entire biosensor microplate every 6 sec, and two scans are averaged to reduce the signal noise, thus leading to a kinetic response with a temporal resolution of ~15 sec. The cultured cells were first washed with the assay buffer HBSS using a plate washer (Bio-Tek Microplate Washers ELx405™, Bio-Tek, Winooski, VT), and incubated inside the Epic® system for ~1 hr. After a steady baseline was established, the ligand solutions were introduced using the on-board liquid handling device, and the cell responses were then recorded over time. For inhibitor profiling, the cells were pretreated with an inhibitor for 1 hr before pinacidil stimulation. For RNAi, the cells were transfected with siRNA for 48 hrs before pinacidil stimulation. All real-time DMR signals were reported as a 2 min baseline, followed by ligand stimulation. All DMR signals were background corrected. At least two independent sets of experiments, each with at least duplicate, were performed.

### Tango β-arrestin translocation gene reporter assay

Tango assays were performed in engineered Tango™ U2OS-GPR35-bla cell line (Life Technologies). This cell line allows an endpoint measure of the activity of agonists specific to the GPR35 activation-induced β-arrestin translocation^69^,^70^. The cells were passed using McCoy's 5A medium supplemented with 10% dialyzed fetal bovine serum, 0.1 μM NEAA, 25 μM Hepes (pH 7.3), 1 mM sodium pyruvate, 100 U/ml penicillin, 100 μg/ml streptomycin, 200 μg/ml zeocin, 50 μg/ml hygromycin, and 100 μg/ml geneticin in a humidified 37°C/5% CO_2_ incubator. For Tango assays, 10000 U2OS-*GPR35-bla* cells per well were seeded in 384-well, black-wall, clear bottom assay plates with low fluorescence background (Corning). After overnight culture, the cells were stimulated with ligands for 5 hrs at 37°C under 5% CO_2_, and then loaded with the cell permeable LiveBLAzer™ FRET (fluorescence resonance energy transfer) B/G substrate. After 2 hr incubation the coumarin to fluorescein ratio was measured using Victor 4 plate reader (PerkinElmer, Waltham, MA, USA). Results obtained were normalized to the maximal response of zaprinast (set to be 100%).

### RhoA activation assay

RhoA activity was determined from protein isolated from C3A cells without and with treatment with 100 μM pinacidil using the luminescence based G-LISATM RhoA activation assay kit (Cytoskeleton, Denver, CO, USA) according to the manufacturer's instructions. Protein was isolated using the provided cell lysis buffer, and cells were processed rapidly on ice and snap-frozen until the time of assaying. Lysates were clarified by centrifugation at 10,000 rpm at 4°C for 2 min. Protein concentration was determined according to the manufacturer's protocol, and cell extracts were equalized to contain protein concentrations of 2 mg/ml for the assay. Luminescence was detected as suggested by the manufacturer.

### Data analysis

All dose-dependent responses were analyzed by using GraphPad Prism 5.0 (GraphPad Software Inc., San Diego, CA, USA). The EC_50_ and IC_50_ values were obtained by fitting the DMR dose response curves with nonlinear regression. For cluster analysis, we profiled the ion channel ligand library across the five cell lines. For effective clustering, we extracted the real responses at the six distinct time points (3, 5, 9, 15, 30, 45 min) for each DMR to form a numerical description of the label-free cell phenotypic characteristics^71^,^72^,^73^. All the six time points refer to the stimulation duration after renormalizing the responses starting from the time when the compound was added. The real responses at these discrete time points were color coded to illustrate relative differences in the direction and strength of a DMR signal. The red color indicates a positive value, while the green color refers to a negative value, the black a value at or near zero. Differences in color intensity illustrate differences in signal strength. In the ligand heatmap matrix (Fig. 1) each column represents one DMR value at a specific time in a specific cell line, and each row represents one ligand. The Ward hierarchical clustering algorithm and Euclidean distance metrics (http://www.eisenlab.org/eisen/) were used for similarity analysis, and every row and column carries equal weight. DMSO in the assay buffer at a concentration that equals to those for all ligands was also included as a negative control. Each DMR represents an average of four replicates. To assist with direct visualization of DMR characteristics of each ligand in a cell line, we did not carry out similarity analysis among distinct columns.

### Statistical analysis

DMR data were analyzed by using GraphPad Prism 5.0 (GraphPad Software Inc., San Diego, CA, USA). The EC_50_ values were obtained by fitting the dose DMR response curves with nonlinear regression. The RNAi effect was analyzed using one-way ANOVA Tukey analysis with the Prism 5.0.

## Author Contributions

H.S. conducted the most DMR assays, RNAi knockdown, RT-PCR, Western blotting, electrophysiology, RhoA activation assay and analyzed the data. Y.W. conducted part of DMR assays, and analyzed the data. H.D. conducted GPR35 studies, and analyzed the data. Q.X. conducted electrophysiology experiments. H.S., M.L., J.L. and Y.F. conceived the idea. Y.F. designed experiments, developed analysis tool, analyzed the data, and wrote the manuscript.

## Supplementary Material

Supplementary Information Supplementary Materials
