# Supplementary material for: Label-free cell phenotypic profiling decodes the composition and signaling of an endogenous ATP-sensitive potassium channel
Source: Sci Rep. 2014 May 12;4:4934. doi: 10.1038/srep04934 (PMC4017216; doi:10.1038/srep04934)
Supplement: Supplementary Information — Supplementary Materials [file srep04934-s1.doc]

**Supplementary Materials**

Label-free cell phenotypic profiling decodes the composition and signaling of an endogenous ATP-sensitive potassium channel

Haiyan Sun^a,+^, Ying Wei^a^, Huayun Deng^a^, Qiaojie Xiong^b,ζ^, Min Li^b^, Joydeep Lahiri^a^, and Ye Fang^a,**^

^a^Biochemical Technologies, Science and Technology Division, Corning Incorporated, Corning, NY 14831, United States of America

^b^The Solomon H. Snyder Department of Neuroscience and High Throughput Biology Center, Johns Hopkins University School of Medicine, Baltimore, Maryland 21205, United States of America

^+^Present address: Biodesign Institute, Arizona State University, AZ 85287, USA

^ζ^Present address: Cold Spring Harbor Laboratory, Cold Spring Harbor, NY 11724, United States of America

** Correspondence and requests for materials should be addressed to Y.F. ([fangy2@corning.com](mailto:fangy2@corning.com))

**Supplementary Figure S1**. Original western blot of of C3A cell lysates using anti-JAK2 for cell lysate without (-) and with (+) 100µM pinacidil treatment (see Figure 7d).

**Supplementary Figure S2**. Original western blot of C3A cell lysates using anti-JAK3 for the lysate of mock transfected cells without (-) and with (+) 100µM pinacidil treatment (see Figure 7e).

**Supplementary Figure S3**. Original western blot of C3A cell lysates using anti-JAK2 for the lysate of Kir6.2 siRNA treated cells without (-) and with (+) 100µM pinacidil treatment (see Figure 7f).

**Supplementary Figure S4**. Original western blot of C3A cell lysates using anti-ROCK1 for cell lysate of untransfected, mock transfected, or ROCK1 siRNA treated cells (see Figure 8d).

**Supplementary Figure S5**. Original western blot of C3A cell lysates using anti-ROCK2 for cell lysate of untransfected, mock transfected, or ROCK1 siRNA treated cells (see Figure 8e).

**Supplementary Figure S1**. Original western blot of of C3A cell lysates using anti-JAK2 for cell lysate without (-) and with (+) 100µM pinacidil treatment. (a) anti-JAK2; (b) anti-actin (see Figure 7d).

**Supplementary Figure S2**. Original western blot of C3A cell lysates using anti-JAK3 for the lysate of mock transfected cells without (-) and with (+) 100µM pinacidil. (a) anti-JAK3; (b) anti-actin treatment (see Figure 7e).

**Supplementary Figure S3**. Original western blot of C3A cell lysates using anti-JAK2 for the lysate of Kir6.2 siRNA treated cells without (-) and with (+) 100µM pinacidil treatment. (a) anti-JAK3; (b) anti-actin (see Figure 7f).

**Supplementary Figure S4**. Original western blot of C3A cell lysates using anti-ROCK1 for cell lysate of untransfected, mock transfected, or ROCK1 siRNA treated cells. (a) anti-ROCK1; (b) anti-actin (see Figure 8d).

**Supplementary Figure S5**. Original western blot of C3A cell lysates using anti-ROCK2 for cell lysate of untransfected, mock transfected, or ROCK1 siRNA treated cells. (a) anti-ROCK2; (b) anti-actin (see Figure 8e).
